# Traumatic Dorsal Dislocation of the Trapezium: Surgical Management of a Rare Hand Injury

**DOI:** 10.7759/cureus.105318

**Published:** 2026-03-16

**Authors:** Gladys Montserrat Ballesteros-Solís, Jorge Alberto García-Garza, José Luis Villarreal-Salgado, Gerardo Salvador Rea-Martínez, José de Jesús Vargas-Montes

**Affiliations:** 1 General Surgery, Instituto de Seguridad y Servicios Sociales de los Trabajadores del Estado (ISSSTE), Guadalajara, MEX; 2 General Surgery, Instituto de Seguridad y Servicios Sociales de los Trabajadores del Estado (ISSSTE), Monterrey, MEX; 3 Plastic Surgery, Instituto de Seguridad y Servicios Sociales de los Trabajadores del Estado (ISSSTE), Guadalajara, MEX; 4 Plastic Surgery, Instituto de Seguridad y Servicios Sociales de los Trabajadores del Estado (ISSSTE), Zapopan, MEX

**Keywords:** carpal instability, hand surgery, kirschner wire fixation, open reduction, trapezium dislocation

## Abstract

Isolated dorsal dislocation of the trapezium is an exceptionally rare carpal injury, typically secondary to high-energy trauma, and carries a high likelihood of being overlooked during the initial radiographic evaluation. Its identification requires careful assessment of carpal alignment, particularly the Gilula arcs, as radiographic alterations may be subtle. Trapezial stability depends on a complex capsuloligamentous system, with the dorsoradial ligament playing a key role; disruption of this structure permits dorsoradial displacement of the bone and results in joint instability. We present the case of a 58-year-old male who sustained high-energy trauma following a motorcycle accident, with direct impact to the left hand. Eight days later, he sought medical evaluation. AP and oblique radiographs demonstrated carpometacarpal dislocation with dorsal displacement of the trapezium, without apparent associated fractures. Given the observed instability, a dorsal surgical approach was performed, followed by open reduction and stabilization using three Kirschner wires. The immediate postoperative course demonstrated adequate restoration of articular congruity and satisfactory carpal alignment. The literature indicates that delayed diagnosis may hinder anatomical reduction due to perilesional fibrosis and increase the likelihood of salvage procedures, such as trapeziectomy, with potential functional consequences. Early recognition and prompt surgical stabilization allow preservation of the trapezium, restoration of thumb biomechanics, and reduction of the risk of chronic instability and post-traumatic osteoarthritis.

## Introduction

Trapezium dislocations constitute an uncommon carpal injury, particularly when they occur in the absence of associated fractures [1]. Their clinical presentation is typically related to high-energy trauma, such as motorcycle accidents (the leading cause reported in published case series), falls, or crush mechanisms capable of generating axial loads and hyperflexion forces [1,2].

The diagnostic challenge lies in the fact that these injuries do not always produce obvious alterations in conventional carpal morphology on plain radiographs, thereby requiring meticulous interpretation of the carpal alignment lines originally described by Gilula in 1979 [2]. Disruption of the extended Arc I between the scaphoid and the trapezium represents a critical radiographic finding for identifying dislocation in both the radioulnar and volar-dorsal planes [2].

From a biomechanical standpoint, trapezial stability depends on a complex capsuloligamentous network composed of multiple ligaments, among which the dorsoradial ligament plays a key role; its disruption allows dorsoradial displacement of the bone [3,4]. The rarity of this entity, its potential to progress to chronic instability, and the possibility of being overlooked on initial imaging make it a significant diagnostic and therapeutic challenge for the hand surgeon [4]. Therefore, we present the following case of dorsal trapezium dislocation, a scarcely documented entity, including its surgical management and immediate postoperative follow-up.

## Case presentation

**Figure 1:**
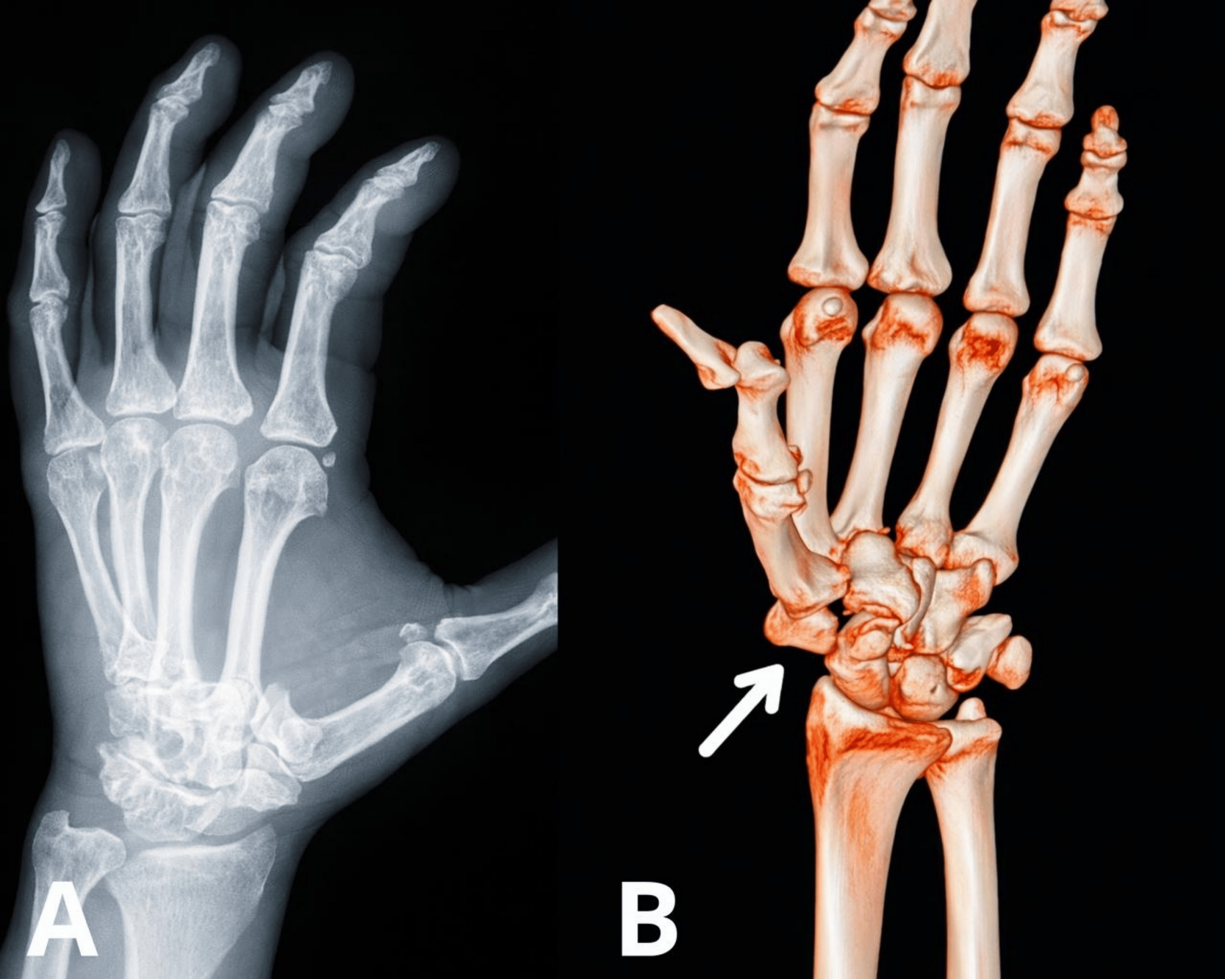
Imaging studies demonstrating trapeziometacarpal dislocation (A) AP radiograph of the hand. (B) CT scan with 3D reconstruction.

A 58-year-old male presented eight days after sustaining an injury in a motorcycle accident, resulting in high-impact trauma to the left hand. He was evaluated in the emergency department, where AP and oblique hand radiographs, as well as CT with 3D reconstruction, were performed. Imaging revealed a carpometacarpal dislocation with displacement of the trapezium (Figure 1A, 1B).

Initial management consisted of immobilization with a thumb spica splint, maintaining the wrist in slight extension (approximately 20-30°), the thumb in radial abduction with slight flexion at the metacarpophalangeal joint, and the interphalangeal joint in a neutral position. Surgical intervention was deemed necessary to achieve stabilization of the dislocation.

**Figure 2:**
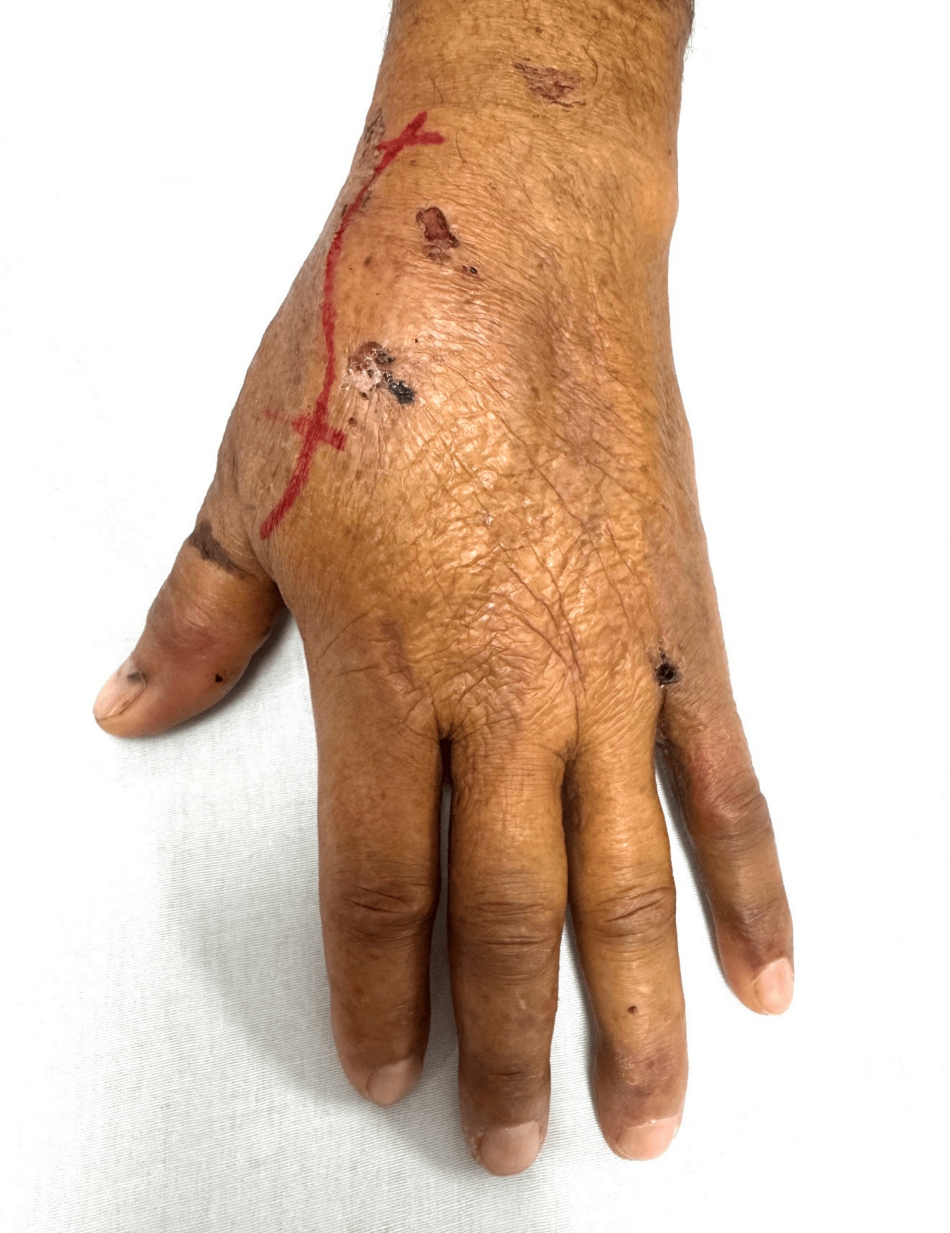
Preoperative anatomical marking Preoperative skin marking outlining the anatomical landmarks of the first carpometacarpal joint, including the base of the first metacarpal and the trapezium.

**Figure 3:**
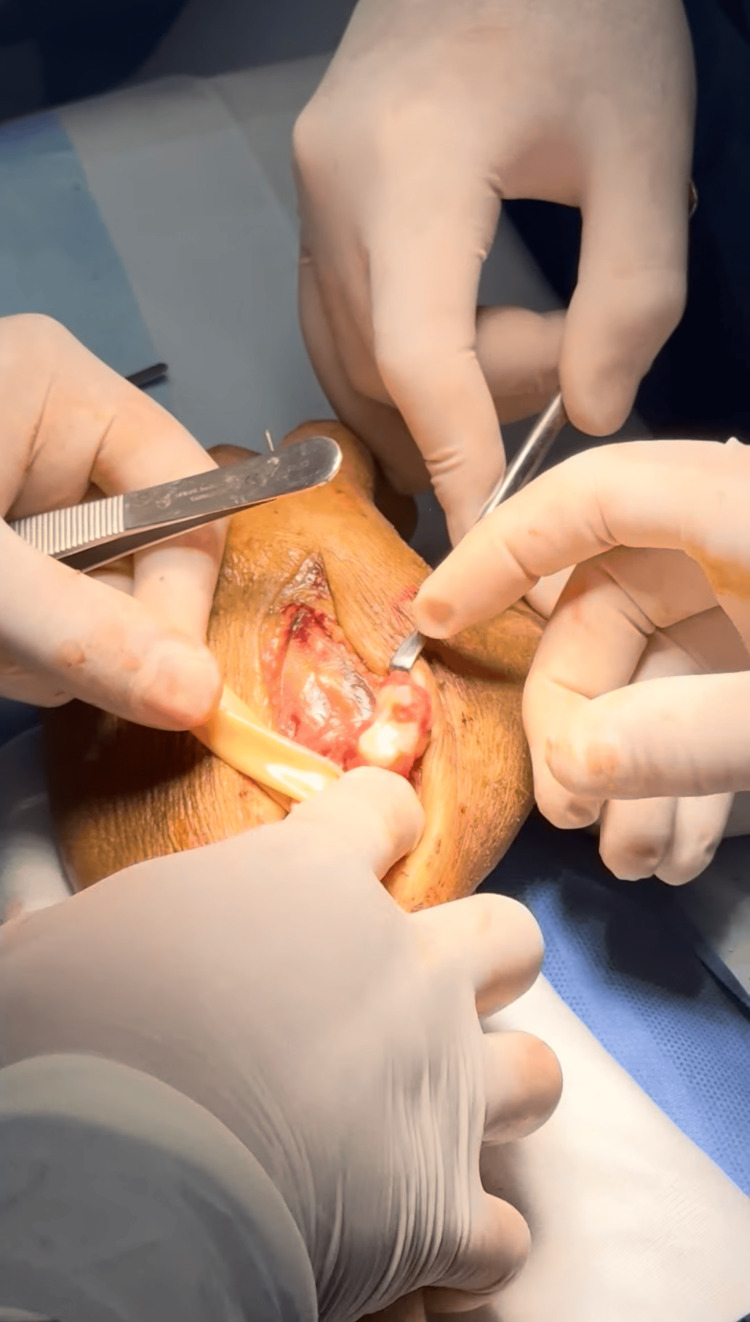
Trapeziometacarpal dislocation Intraoperative view of the trapeziometacarpal dislocation showing displacement of the trapezium relative to the base of the first metacarpal.

Following anatomical landmark marking (Figure 2), a dorsal surgical approach was performed through an incision over the dorsal aspect of the left hand. Layered dissection revealed intact extensor tendons, and upon reaching the osseous structures, a dorsal trapeziometacarpal dislocation was confirmed (Figure 3).

**Figure 4:**
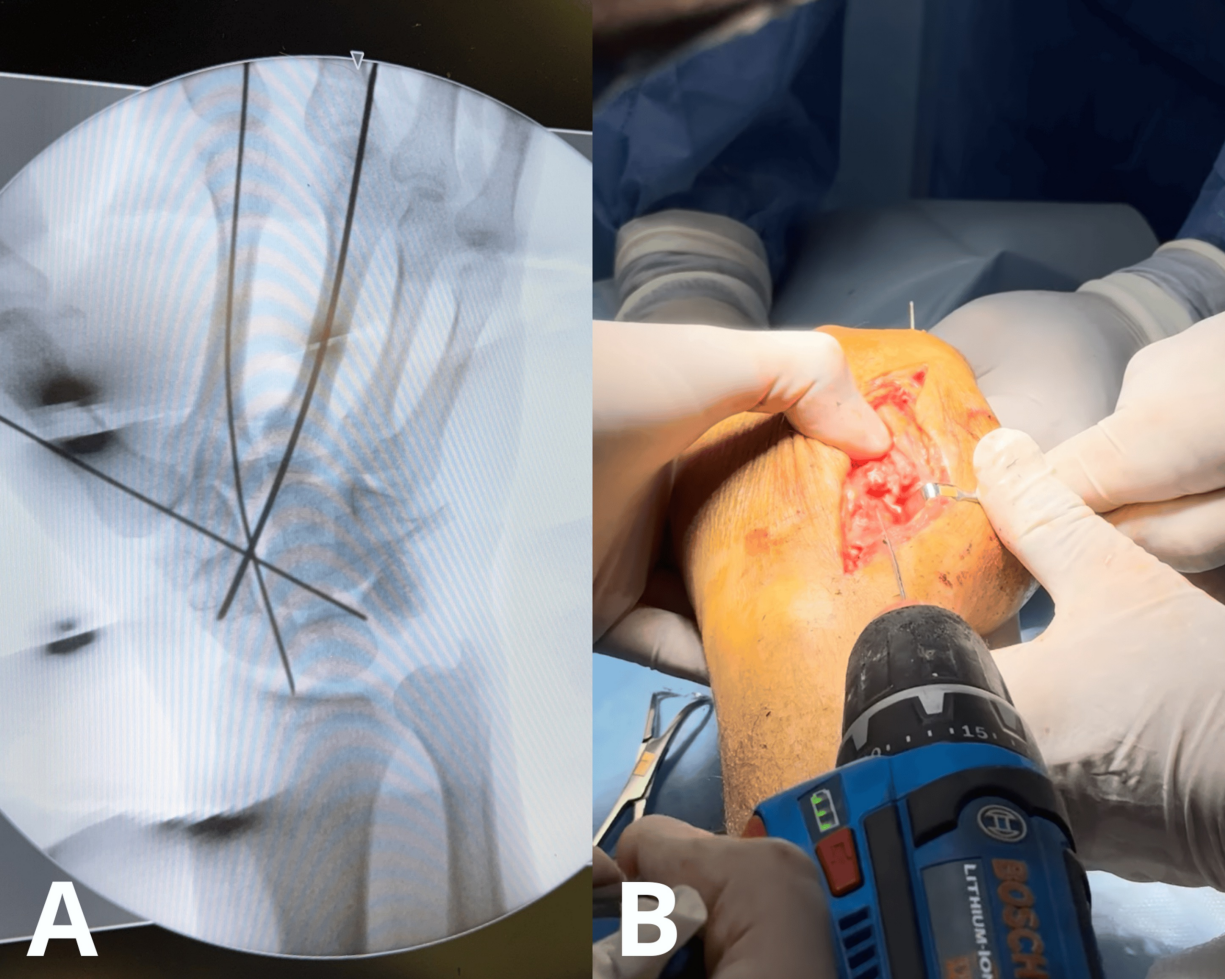
Intraoperative Kirschner wire fixation (A) Intraoperative radiograph after placement of Kirschner wires demonstrating stabilization of the trapeziometacarpal joint. (B) Placement of Kirschner wires to restore proper alignment of the carpometacarpal joint.

Subsequently, open reduction of the dislocation was performed, and three 1.2-mm Kirschner wires were placed for stabilization, ensuring restoration of articular congruity and proper alignment of the carpometacarpal joint (Figure 4A, 4B).

In the postoperative period, the patient demonstrated an appropriate clinical course without acute complications related to the procedure. The hand was immobilized with a thumb spica splint to maintain carpometacarpal stability. He was discharged two days after surgery with prescriptions for analgesics and antibiotics.

At the subsequent postoperative evaluation, seven days later, the surgical wound had well-approximated edges without signs of local complications. The Kirschner wires were removed four weeks after surgery once clinical and radiographic stability was confirmed. Following wire removal, the patient began a physiotherapy program focused on gradual restoration of wrist and thumb range of motion, followed by progressive strengthening exercises to recover functional mobility.

## Discussion

The direction of trapezial displacement constitutes a key element in understanding the magnitude of the associated capsuloligamentous injury. Several reports indicate that failure of closed reduction may be attributed to capsular interposition, osteochondral adhesions, or rotational displacement of the trapezium, factors that justify open reduction when instability persists after attempted closed maneuvers [1]. Furthermore, delayed diagnosis promotes pericapsular fibrosis and complicates anatomical restoration, particularly when the injury is not recognized on initial radiographs, a scenario classically emphasized in the systematic evaluation of carpal alignment [2].

Previous literature also suggests that early detection through advanced imaging studies can reduce the need for ablative procedures and improve functional outcomes [3,4]. When treated early, functional recovery is generally favorable, with return to work reported at approximately eight weeks and satisfactory thumb mobility [1]. Comparable outcomes have been described in contemporary case series when stable anatomical reduction and early, structured rehabilitation protocols are implemented [4,5].

Dorsoradial dislocations are typically related to disruption of the dorsoradial ligament, a structure whose injury has been associated with significant loss of trapezial stability in clinical reports [6,7]. In contrast, volar dislocations, which have been reported more frequently than pure radial dislocations, are commonly described following direct impact or crush mechanisms and may be associated with increased tension on the volar ligamentous complex, with potential involvement of the recurrent branch of the median nerve [8,9].

From a biomechanical standpoint, the dorsoradial ligament complex functions as a primary stabilizer against dorsoradial subluxation of the trapeziometacarpal joint, as demonstrated in cadaveric models evaluating ligamentous restraints [10]. These displacement patterns are not merely descriptive but have therapeutic implications, as they influence the ease of reduction and the need for additional stabilization, reflecting the degree of ligamentous disruption and the energy transmitted to the distal carpus [4].

Once anatomical reduction has been achieved, temporary transosseous fixation plays a determinant role in maintaining stability. The combination of scaphotrapezoid and trapeziotrapezoid Kirschner wire fixation has been reported to provide effective neutralization of deforming forces generated by the thenar musculature, particularly in cases associated with rotational displacement [4]. In acute injuries, percutaneous fixation with Kirschner wires has demonstrated favorable functional outcomes when adequate stability is obtained after reduction, whereas open reduction is generally reserved for irreducible or unstable dislocations [1,4].

Delayed diagnosis remains one of the main determinants of prognosis. Progressive perilesional fibrosis significantly reduces the likelihood of achieving anatomical reduction and may necessitate salvage procedures such as trapeziectomy in chronic cases [1,4]. Although this procedure may provide pain relief, it has been associated with decreased pinch strength, proximal migration of the first metacarpal, and long-term functional limitations [8,9].

Overall, the available literature supports a management strategy focused on preserving the trapezium whenever feasible, complemented by temporary stabilization and early guided rehabilitation [3-5]. In the present case, stable fixation with Kirschner wires combined with postoperative thumb spica immobilization followed by structured physiotherapy allowed satisfactory recovery without complications. However, the current evidence is largely based on case reports and small case series, highlighting the need for continued documentation of these uncommon injuries to establish more standardized therapeutic criteria and optimize long-term functional outcomes [4,10].

## Conclusions

Isolated trapezium dislocation is a rare but potentially disabling injury that requires a high index of suspicion for timely diagnosis. Because its clinical presentation may be subtle and easily overlooked, careful physical examination and appropriate imaging studies are essential to avoid missed or delayed identification. Early recognition is critical to prevent chronic instability and long-term functional impairment.

Treatment should be individualized based on the timing of presentation and post-reduction stability. In acute cases, prompt anatomical reduction and temporary stabilization can preserve joint congruity and lead to satisfactory functional recovery. Delayed diagnosis, however, increases technical difficulty and may necessitate salvage procedures associated with reduced pinch strength and residual functional limitations.
